# Fusarium head blight resistance exacerbates nutritional loss of wheat grain at elevated CO_2_

**DOI:** 10.1038/s41598-021-03890-9

**Published:** 2022-01-07

**Authors:** William T. Hay, James A. Anderson, Susan P. McCormick, Milagros P. Hojilla-Evangelista, Gordon W. Selling, Kelly D. Utt, Michael J. Bowman, Kenneth M. Doll, Kim L. Ascherl, Mark A. Berhow, Martha M. Vaughan

**Affiliations:** 1 Mycotoxin Prevention and Applied Microbiology Unit, Agricultural Research Service, USDA, National Center for Agricultural Utilization Research, 1815 N, University Street, Peoria, IL 61604 USA; 2 Department of Agronomy and Plant Genetics, University of Minnesota, St. Paul, MN 55108 USA; 3 Plant Polymer Research Unit, Agricultural Research Service, USDA, National Center for Agricultural Utilization Research, 1815 N, University Street, Peoria, IL 61604 USA; 4 Bioenergy Research Unit, Agricultural Research Service, USDA, National Center for Agricultural Utilization Research, 1815 N, University Street, Peoria, IL 61604 USA; 5 Bio-Oils Research Unit, Agricultural Research Service, USDA, National Center for Agricultural Utilization Research, 1815 N, University Street, Peoria, IL 61604 USA; 6 Functional Foods Research Unit, Agricultural Research Service, USDA, National Center for Agricultural Utilization Research, 1815 N, University Street, Peoria, IL 61604 USA

**Keywords:** Climate-change impacts, Abiotic, Plant immunity, Plant sciences

## Abstract

The nutritional integrity of wheat is jeopardized by rapidly rising atmospheric carbon dioxide (CO_2_) and the associated emergence and enhanced virulence of plant pathogens. To evaluate how disease resistance traits may impact wheat climate resilience, 15 wheat cultivars with varying levels of resistance to Fusarium Head Blight (FHB) were grown at ambient and elevated CO_2_. Although all wheat cultivars had increased yield when grown at elevated CO_2_, the nutritional contents of FHB moderately resistant (MR) cultivars were impacted more than susceptible cultivars_._ At elevated CO_2_, the MR cultivars had more significant differences in plant growth, grain protein, starch, fructan, and macro and micro-nutrient content compared with susceptible wheat. Furthermore, changes in protein, starch, phosphorus, and magnesium content were correlated with the cultivar FHB resistance rating, with more FHB resistant cultivars having greater changes in nutrient content. This is the first report of a correlation between the degree of plant pathogen resistance and grain nutritional content loss in response to elevated CO_2_. Our results demonstrate the importance of identifying wheat cultivars that can maintain nutritional integrity and FHB resistance in future atmospheric CO_2_ conditions.

## Introduction

Wheat is the most cultivated crop world-wide and accounts for nearly one fifth of all human dietary protein^1,2^. The nutritional integrity of wheat is endangered by rapidly rising atmospheric carbon dioxide (CO_2_) and the associated emergence and enhanced virulence of plant pathogens^3–5^. Grain protein and mineral content decline at elevated CO_2_ in major C3 photosynthetic cereal crops such as wheat, negatively impacting end-use quality and ultimately food security^5–8^. Furthermore, climate change is predicted to increase the risk of mycotoxin contamination associated with fungal diseases such as Fusarium Head Blight (FHB)^9^, and elevated CO_2_ may provide a strain-specific pathogenic advantage on hosts with greater losses in nutritional content^10^. FHB outbreaks can substantially diminish grain yield and end-use quality due to sterile florets and withered, mycotoxin contaminated grain kernels^11^. Cultivating wheat germplasm that are climate resilient and possess some resistance to FHB is a key control strategy for maintaining food safety and security.

Previous studies have shown that wheat grown at elevated CO_2_, with sufficient water and fertilization, will have substantially increased yields but the grain will contain higher carbohydrate content and lower relative amounts of proteins, minerals, and lipids^12–14^. This effect, often referred to as dilution, is generally caused by an excess accumulation of carbohydrates from enhanced photosynthetic carbon metabolism; however, disproportionate losses in specific nutrients indicate a more complex biological partitioning mechanism may be partially responsible^6,13,15,16^. Carbohydrate dilution of grain protein results in wheat flour with reduced baking quality, limiting the end-use utility and producing less nutritious, lower value food goods^7,17,18^.

The impact of elevated CO_2_ on grain quality is cultivar dependent, and even known adaptive traits, such as high nitrogen use efficiency and improved root vigor, are not sufficient to counteract the effects on grain protein and recover end-use quality^19,20^. The effects of rising CO_2_ not only impacts wheat nutritional content but can also increase plant disease susceptibility, particularly to FHB^3,21–23^. The combined adverse effects of elevated CO_2_ on wheat nutritional content and disease susceptibility is a significant threat to wheat producers and consumers. Currently, plant differential response to elevated CO_2_ has been studied in a very limited number of cultivars and crops; greater screening efforts could uncover meaningful differences in elevated CO_2_ response, which could be exploited by plant breeders^12^. It is vitally important to determine whether FHB resistance is associated with greater grain nutritional losses at elevated CO_2_ and to identify climate and FHB resilient cultivars.

In spring wheat, moderate resistance to FHB is predominantly derived from the genetic background of the Sumai 3 cultivar, largely due to the *Fhb1* Quantitative Trait Locus (QTL). Although, there are other sources of resistance (*Fhb2, Fhb4, Fhb5, Fhb7*), there is no combination of these, or other genes, that provide complete resistance to FHB^24^. While individual QTLs provide a degree of resistance, the introgression of *Fhb1, Fhb2,* and *Fhb5,* as well as the stacking of two or more FHB QTLs, has been associated with reduced grain protein content^25,26^. We recently observed that the difference in grain protein content between wheat grown at 400 ppm CO_2_ versus 800 ppm was significantly greater in the FHB moderately resistant hard red spring wheat cultivar Alsen, which contains the *Fhb1* and *Fhb5* loci, in comparison to the susceptible cultivar Norm, which does not^10^. Consequently, we investigated whether FHB resistance factors from the Sumai 3 background could impact grain nutritional content in future atmospheric conditions.

Based on our results with the wheat cultivars Alsen and Norm, we hypothesized that the direct effects of elevated CO_2_ more severely impact the grain composition of FHB moderately resistant wheat cultivars than susceptible cultivars. To test this hypothesis, six FHB susceptible cultivars and nine moderately resistant cultivars (Table 1) were grown in triplicate experiments at ambient 400 ppm (a[CO_2_]) or elevated 1000 ppm (e[CO_2_]), and then evaluated for differences in plant development, physiology, yield, and nutritional composition. Differences between wheat cultivars and FHB resistance scores (Table 1) were identified to evaluate whether the presence of FHB resistance genes will be a nutritional liability for wheat cultivars in the future.

## Materials and methods

### Wheat cultivars and growth conditions

FHB susceptible hard red spring wheat cultivars with FHB scores ranging from 5 to 9, Linkert^27^, MN00269, MN10281-1-98, MN11492-6, Ulen^28^, Wheaton^29^, and FHB moderately resistant (MR) cultivars with FHB scores ranging from 2 to 4, (Bolles^30^, Glenn^31^, Lang-MN^32^, MN08172-3-10, MN11394-6, RB07^33^, Rollag^34^, Sabin^35^, Shelly^36^) (Table 1), were grown in controlled environment growth chambers according to the methods outlined in Hay, et al.^10^. The relative FHB resistance of wheat cultivars is based on evaluations in 6 or more inoculated, misted field environments for FHB traits: incidence, severity, Visual Scabby Kernels, and DON contamination. New cultivars are compared against cultivars with an established 1–9 FHB rating for classification^32^. For the cultivation and harvest of wheat, all local and national regulations were followed, and all relevant permissions were acquired. No genetically modified plant cultivars were examined in the study.

**Table 1:** Breeding pedigrees for wheat cultivars in the current study.

Moderately resistant	FHB resistance score	*Fhb1* QTL	Pedigree
MN08173-3-10	2^a^	+	MN01261-8-1/MN03111-3
Glenn	3		ND 2831/ ‘Steele-ND’ (PI 634981)
Lang MN	3	+	Glenn/Sabin
MN11394-6	3	+	MN00209-3-1/MN05209
Rollag	3	+	MN95229‐40*2/RL4970‐4
Bolles	4		MN02268-1/MN01333-A-1
RB07	4	+	‘Norlander’(PI 591623)/ ‘HJ98’
Sabin	4	+	MN98389/MN97518
Shelly	4	+	‘Faller’//00H04*J3/MN03130-1-62
**Susceptible**			
Linkert	5		MN97695-4/ ‘Ada’ sel
MN10281-1-98	6		MN02072-7/Faller
MN11492-6	6		RB07/Blade
Ulen	6		MN92044/HJ98
MN00269	9		MN2450W/MN94346
Wheaton	9		Crim/2*Era//Buitre/Gallo

Resistance to *Fusarium* head blight (FHB) scored on a 1–9 scale (1 fully resistant, 9 highly susceptible), based off of FHB nursery field evaluations. + indicates the presence of the *Fhb1* QTL in a cultivar. ^a^Indicates preliminary FHB resistance score; cultivar is currently undergoing multi-year field trials. All cultivars were provided by Dr. James Anderson except for Glenn, which was purchased from a local seed provider. Of the MR cultivars tested two lack *Fhb1*, Glenn and Bolles. Unlike the other MR cultivars, Bolles is more distantly related to Sumai 3 (Zhu et al.^24^; Anderson et al.^27,30^; Mergoum et al.^31^), but Bolles’ great-grandparent Nyu Bai is of Japanese origin and has several FHB resistance QTLs that are related to other Chinese sources of resistance like Sumai 3 (McCartney et al.^26^; Somers et al. 2003).

The growth chambers were blocked into pairs, with each block containing a chamber set to ambient [CO_2_] (420 ± 20 ppm, a[CO_2_]) and a chamber set to 1000 ± 20 ppm [CO_2_] (e[CO_2_]). Experiments were performed in triplicate between 2018 and 2019. The wheat cultivars were grown in a completely random block design. For each cultivar, five plants were grown in a 20 × 15-cm plastic pot, filled with approximately 4 L of SunGrow Horticulture potting mix (Agawam, MA, U.S.A.). Growth chambers were programmed at 25/23 °C day/night, respectively, a 14 h photoperiod at 550 μmol m^−2^ s^−1^ photosynthetic photon flux density, and 50–60% relative humidity. Plants were watered daily and were fertilized biweekly with soluble Peters 20–20–20 nutrient supplement (The Scotts Company, Marysville, OH, U.S.A.) until anthesis. Plant positions were randomized after each watering. The developmental timings of heading (Feekes 10.2) and flowering (Feekes 10.5.2) were monitored, and tiller number and height were measured once plants reached physiological maturity (Feekes 11.3). After ripening (Feekes 11.4), grain was harvested for yield and compositional evaluations. Wheat grain moisture and protein were determined by near-infrared spectroscopy (NIR), using a DA 7250 NIR analyzer (Perten Instruments, Springfield, IL). Samples were then milled into whole wheat flour using a Retsch ZM200 ultra centrifugal mill (Retsch, Haan, Germany), equipped with a 12-tooth stainless steel rotator spinning at 10,000 rpm with a 0.75 mm stainless steel screen.

### Carbohydrate analysis

For each growth experiment, each sample was prepared in triplicate (i.e. 3 replicates × 3 growth experiments × 15 cultivars × 2 [CO_2_] = 270 samples). Water soluble carbohydrate content (WSC) for each sample was determined in triplicate by high-performance anion-exchange chromatography-pulsed amperometric detection (HPAEC-PAD), following the protocol outlined in Hay, et al.^10^. Grain starch and fructan content was determined in triplicate by using a sequential procedure as outlined by Dien, et al.^37^. Grain soluble carbohydrates were extracted from 100 mg of the whole wheat flour with 5 mL of 80% v/v ethanol at 60 °C for 1 h. Samples were then transferred to a 4 °C refrigerator and stored for 16–22 h before centrifugation at 1925× g for 15 min. The fructan concentration of the supernatant was determined by hydrolyzing samples to fructose for colorimetric measurement of monomeric fructose^38^. To hydrolyze starch to glucose, the alcohol-insoluble residue was treated with 6 µg, (3.5 Units where one unit will liberate 1.0 mg of maltose from starch in 3 min at pH 6.9 at 20 °C) of heat-stable α-amylase (from *Bacillus licheniformis*, Sigma-Aldrich, Saint Louis, MO) for 1 h at 90 °C in 5 mL of 0.1 M acetate buffer, pH 5. The sample was subsequently treated with 600 µg (8.6 Units where one unit will liberate 1.0 mg of glucose from starch in 3 min at pH 4.5 at 55 °C.) amyloglucosidase (from *Aspergillus niger*, Roche, Mannheim, Germany) for 3 h at 60 °C. Finally, 20 mL of each sample was analyzed in by a high-performance liquid chromatography (Ultimate 3000 (Thermo Scientific, Waltham, MA)) for glucose using a refractive index detector through an Aminex-87H column running 5 mM sulfuric acid isocratically at 0.6 mL/min flow rate at 60 °C. Sample temperature was maintained at 4 °C in the autosampler prior to injection.

### Fatty acid analysis

The ratios of grain fatty acids were determined by hydrolysis and fatty acid methyl ester formation modified from the procedure of Hartman and Lago^39^, and analysis by gas chromatography mass spectrometry (GC–MS) in triplicate. Briefly, 60 mg of whole wheat flour was treated with 1 mL of 0.25 M sodium methoxide at 60 °C for 30 min. The sample was cooled to room temperature before 1 mL of hexane and 1 mL of saturated NaCl solution were added and allowed to phase separate without disturbance for 10 min. A 0.5 mL aliquot of the organic layer of each sample was analyzed for fatty acid methyl esters by a Shimadzu GC-2010 Plus GC–MS (Tokyo, Japan), using a capillary column (Supelco SP-2380; 30 m × 0.25 mm). Operating conditions were as follows: Initial oven temperature was set to 130 °C, with a programmed ramp of 20 °C/min to 265 °C which was held for 2.25 min. The split ratio was 100:1 with a column flow rate of 1.2 mL/min and a septum purge rate of 4 mL/min. Injector and detector were set to 250 °C, and hexane was used for control blanks and rinse vials.

### Grain mineral content

Select grain mineral content was determined by inductively coupled plasma-optical emission spectroscopy with a Perkin-Elmer 7000DV ICP (Shelton, CT), according to the protocols outlined in Hay, et al.^10^. All samples were tested in triplicate to determine phosphorous, potassium, magnesium, calcium, manganese, zinc, iron, and copper content.

### Wheat stem physical characteristics

Evaluations of wheat stem physical characteristics were performed following the modified protocols of Miller, et al.^40^. Straw samples from mature wheat plants were equilibrated at 23 °C and 50% RH for one week and cut to lengths of 76 mm from the middle of the 2nd internode region. A 3-Point bend test was conducted on an Instron (Norwood, MA) Model 3300 Controller with a 3365 Frame equipped with a 1 kN load cell using Bluehill Universal software. Samples were centered over supports having a 50.8 mm support span. A circular diameter confirmation was used for the tests. The rate of movement was 10 mm/min with a data sampling rate of 20 ms. Testing was stopped with an event displacement of 25 mm. Internal and external diameters, to determine cross-sectional area, were determined approximately 13 mm from each side of the bend site using a micrometer. The bending stress at material failure was calculated using the Equations^41^:

1 $${\text{Second moment of Inertia:}}\,I =\uppi \left( {{\text{D}}_{{\text{i}}}^{4} - {\text{D}}_{{\text{o}}}^{4} } \right) \, /64$$

2 $${\text{Bending stress at material failure:}}\,\upsigma _{{\text{b}}} = {\text{F}}_{\max } \left( {{\text{R}}_{{\text{o}}} - {\text{R}}_{{\text{i}}} } \right) \, *{\text{span}}/4I$$

where D_i_ is the inner diameter, D_o_ is the outer diameter, F_max_ is the absolute resistance of the stem sample to break under-load, R_o_ is the outer radius, R_i_ is the inner radius, the span of two Instron supports in cm.

### Statistical analyses

Results were evaluated by a generalized linear mixed model analysis of variance, with experimental replicate as a random effect (JMP V15.0), to determine significant differences between cultivars and FHB resistance groups due to the effects of elevated CO_2_ (α = 0.05). Information on pairwise comparisons are detailed within the table and figure legends. Significant correlations between independent variables (α = 0.05) were evaluated by multivariate analysis and a Pearson correlation (*r*) was reported, which ranges from − 1 to 1; where 0 represent no correlation, − 1 represents a perfectly negative correlation, and 1 a perfectly positive correlation. Discriminant analysis was performed using a stepwise variable selection (Smallest *P* to enter ≤ 0.05) and a linear fitting method (JMP V15.0).

## Results

### Effects of elevated CO_2_ on plant development, height, and yield

**Figure 1:**
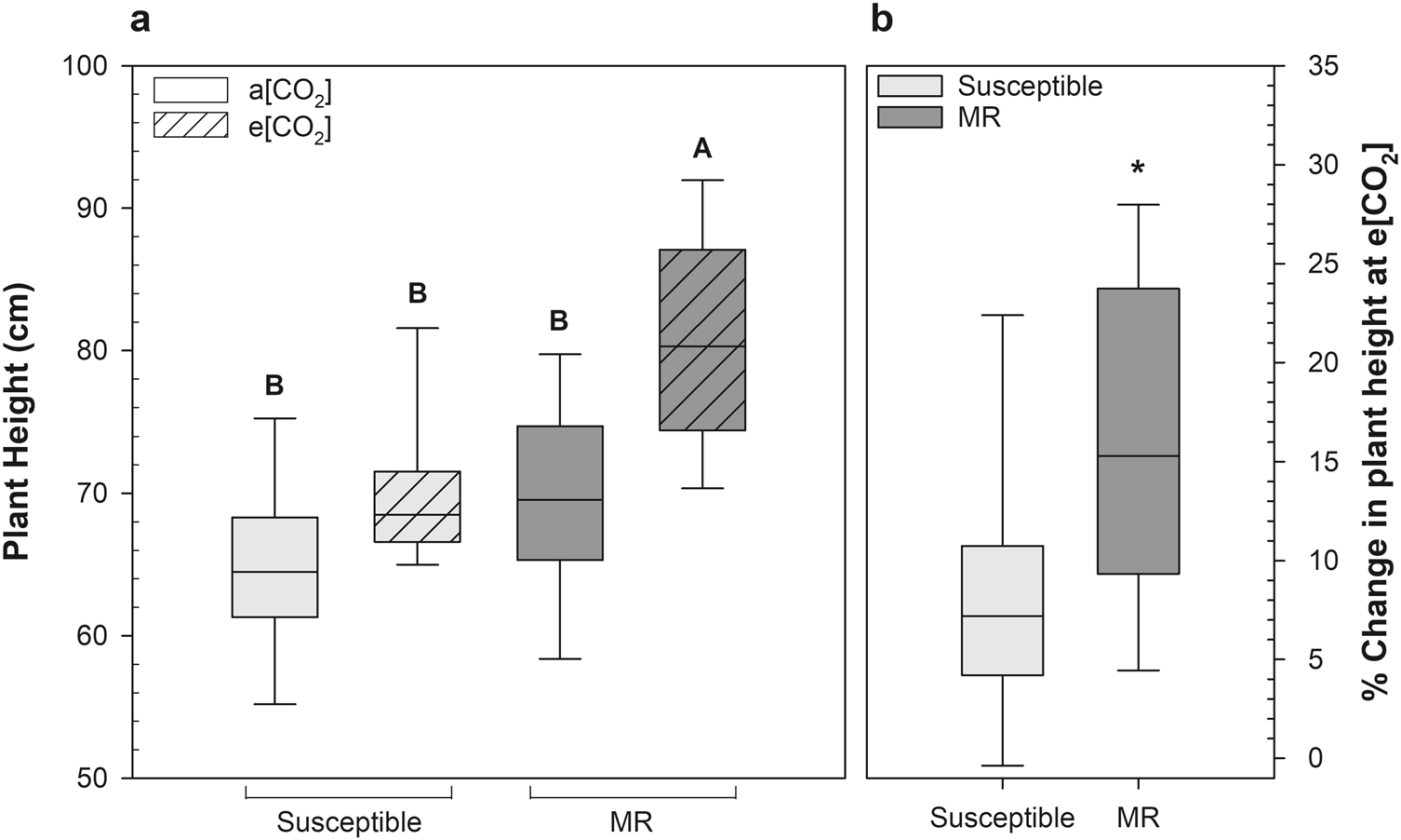
(**a**) Differences in plant height by *Fusarium* head blight (FHB) resistance at ambient CO_2_ (a[CO_2_]) and elevated CO_2_ (e[CO_2_]). (**b**) Growth response of moderately resistant (MR) and susceptible wheat cultivars at e[CO_2_], as determined by differences in plant height at a[CO_2_] compared with the same cultivars grown at e[CO_2_]. Different letters represent statistically significant differences determined by a Tukey HSD analysis of variance (*P* < 0.05); MR (*n* = 27), and susceptible (*n* = 18), for each CO_2_ growth condition. Asterisks (*) denote statistically significant differences (*P* < 0.05).

To determine whether changes in grain composition due to growth at elevated CO_2_ were more severe in FHB MR wheat compared with susceptible, 15 cultivars with varying FHB disease resistance (Table 1) were grown to maturity at a[CO_2_] and e[CO_2_]. Wheat height, stem strength, tiller number, and yield were all significantly impacted by growth at e[CO_2_]. Although plant development was not impacted by elevated CO_2_, wheat cultivars had distinctly different heading and flowering times (supplemental Table 1). The average plant height increased by 9 cm under e[CO_2_] (supplemental Fig. 1); however, the height increases were only statistically significant for the MR group (*P* = 0.043; Fig. 1a).

There was a significant cultivar × [CO_2_] interaction (*P* = 0.016) (supplemental Table 2), and the tallest cultivar, MN08173-3-10, had an average increase in height of 15 cm at e[CO_2_]. The MR cultivars were taller than susceptible cultivars (Fig. 1a) and showed more vigorous vegetative growth at e[CO_2_] (Fig. 1b); the % change in plant height for the MR cultivars was significantly greater than susceptible cultivars (*P* = 0.0002).

Because increases in plant height can negatively impact structural integrity (e.g., lodging), stem strength was evaluated. Stem strength, as determined by bending stress at material failure, was moderately less (6%) in wheat grown at e[CO_2_] (*P* = 0.009), but this was mostly driven by a significant cultivar × [CO_2_] interaction (*P* = 0.0007) and did not correlate with FHB resistance level or difference in plant height at e[CO_2_]. While the stem strength of most cultivars was not negatively impacted by growth at e[CO_2_], the MR cultivars Bolles and RB07 suffered 23% and 25% decreases in stem strength, respectively, at e[CO_2_].

**Figure 2:**
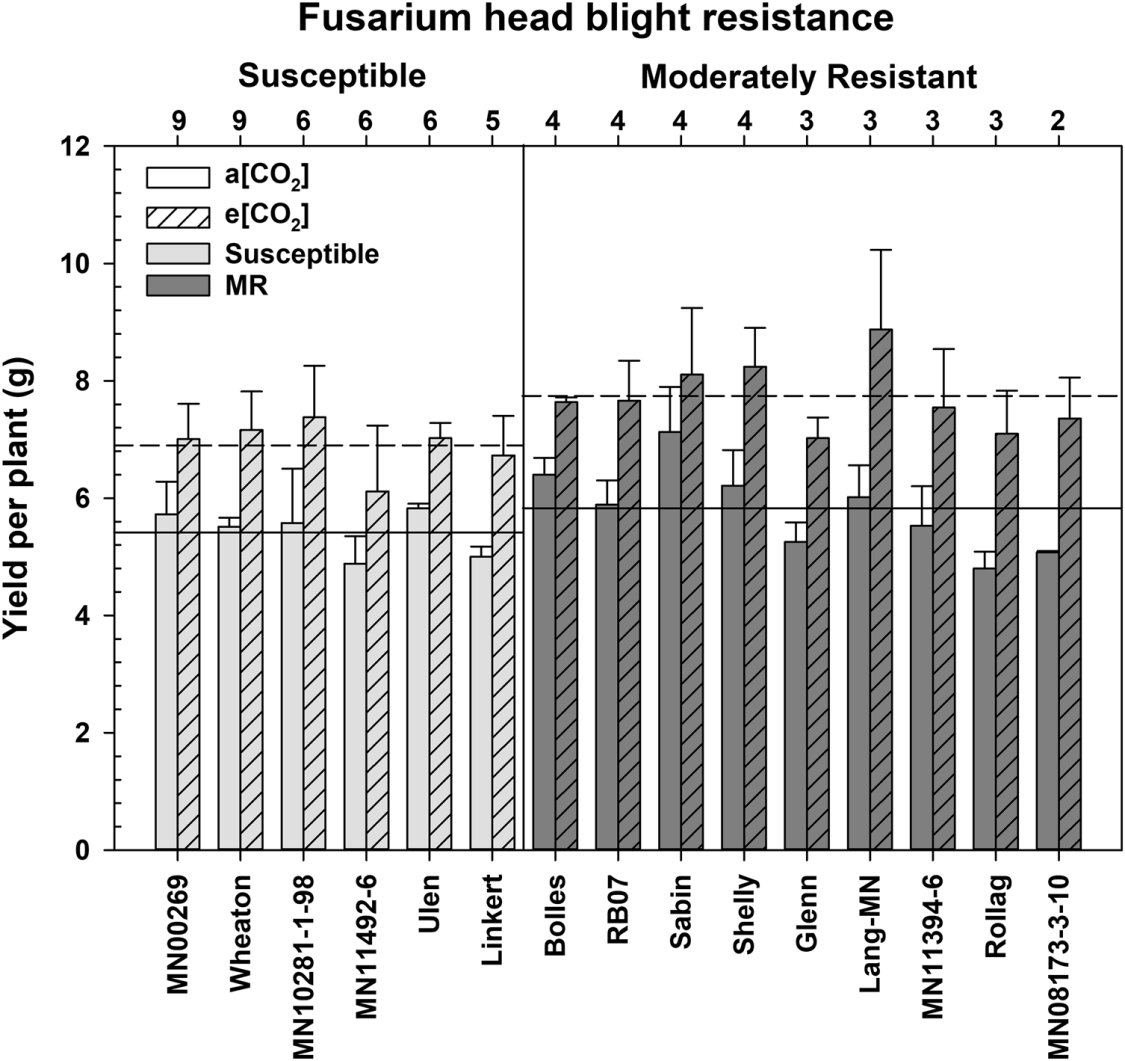
Differences in yield per plant of various *Fusarium* head blight (FHB) moderately resistant (MR) and susceptible wheat cultivars at ambient CO_2_ (a[CO_2_]) and elevated CO_2_ (e[CO_2_]), ordered by FHB resistance score at the top of the figure panel. Solid and dashed lines represent the mean yield per plant for each FHB resistance group at a[CO_2_] and e[CO_2_], respectively.

Though MR wheat was significantly taller than susceptible wheat at e[CO_2_] (Fig. 1b), this did not provide a yield advantage, as each group had an equivalent increase in grain yield at e[CO_2_] (Fig. 2). Grain yield per plant was dramatically higher, by 30%, at e[CO_2_] (*P* < 0.0001) but there was no significant interaction between CO_2_ and cultivar or FHB group. The increased yield was predominately due to an additional tiller per plant at e[CO_2_] (25% increase; *P* < 0.0001); each plant usually had four tillers at a[CO_2_], which increased to five tillers per plant at e[CO_2_]. Yield per tiller had a minor increase of 5% at e[CO_2_] (*P* = 0.043). The average seed weight was not impacted by growth at e[CO_2_], nor significantly different between MR and susceptible groups.

### Effects of elevated CO_2_ on grain composition

**Figure 3:**
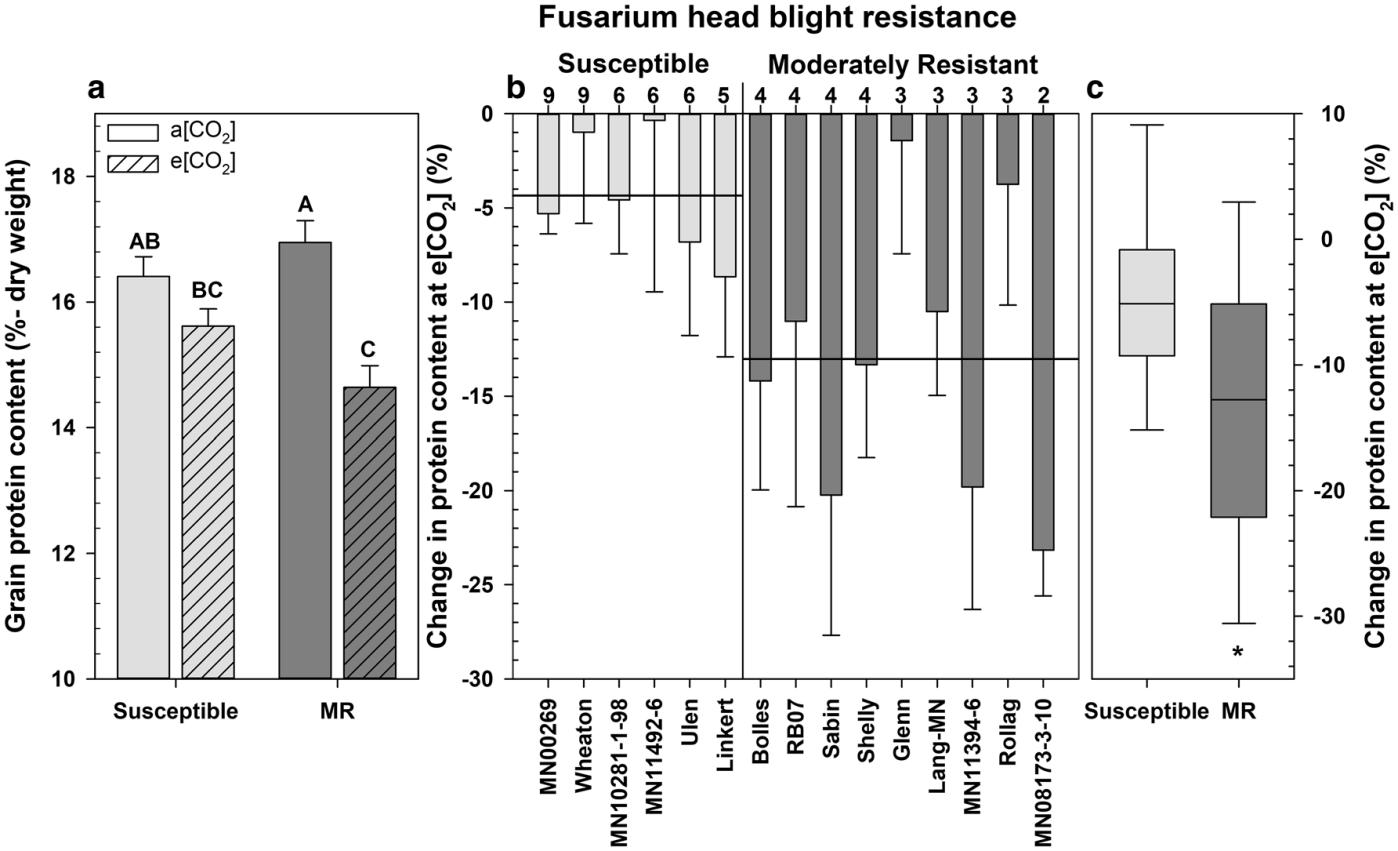
Percent grain protein of various *Fusarium* head blight (FHB) moderately resistant (MR) and susceptible wheat cultivars. Change in protein content determined by evaluating differences in grain protein from wheat grown at elevated CO_2_ (e[CO_2_]), compared with wheat grown at ambient (a[CO_2_]). (**a**) Grain protein content of susceptible and MR wheat grain grown at a[CO_2_] and e[CO_2_], error bars represent standard error. (**b**) Percent change in protein content at e[CO_2_] by cultivar and FHB resistance score. Horizonal bars represent group mean protein loss. Wheat cultivars were ordered by FHB resistance score at the top of the figure panel. (**c**) Percent change in protein content by FHB group. Different letters or asterisks (*) denote statistically significant differences as determined by a Tukey adjusted generalized mixed model analysis of variance (*P* < 0.05); MR (*n* = 27), and susceptible (*n* = 18), for each CO_2_ growth condition.

Growth at e[CO_2_] significantly impacted grain composition, especially the protein content of grain from FHB resistant wheat cultivars (Fig. 3). While there were differences in protein content among cultivars, the cultivar x [CO_2_] interaction was not significant (*P* = 0.07), but there was a significant interaction between the FHB resistance rating × [CO_2_] (*P* = 0.031). On average, there was less protein in both MR and susceptible cultivars at e[CO_2_]_,_ but this difference was only significant for the MR cultivars, which had 13% less protein (*P* = 0.025; Fig. 3a). There was an inverse relationship between the FHB resistance and the change in protein content at e[CO_2_] (*r* = − 0.37; *P* = 0.012; Fig. 3b,c). Furthermore, the overall percent change in protein content at e[CO_2_] was greater in the MR cultivars compared with susceptible (*P* = 0.004; Fig. 3c).

**Figure 4:**
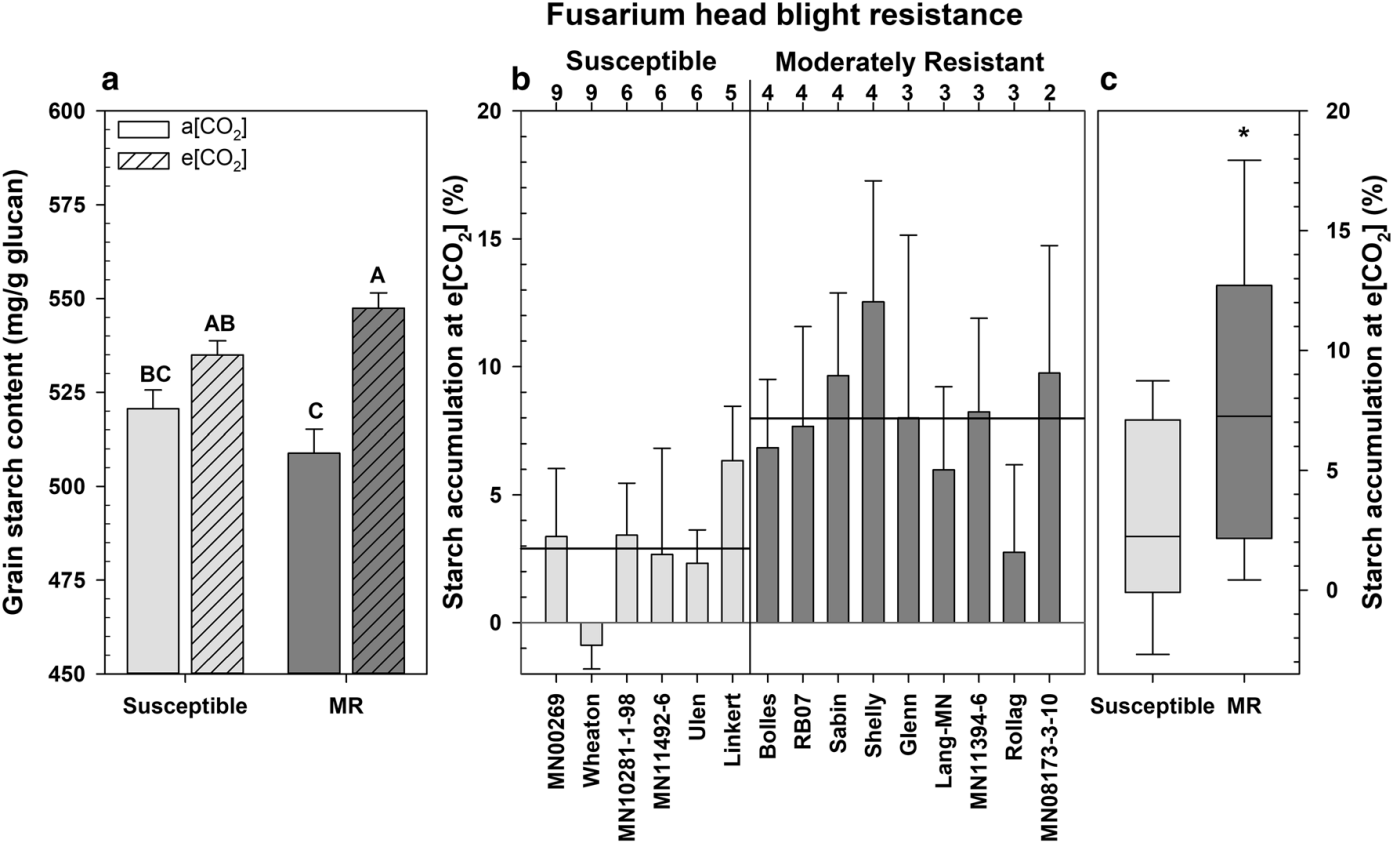
Percent grain starch accumulation of various *Fusarium* head blight (FHB) moderately resistant (MR) and susceptible wheat cultivars. Starch accumulation determined by evaluating differences in starch content from wheat grown at elevated CO_2_. (**a**) Grain starch content of susceptible and MR wheat grain grown at ambient (a[CO_2_]) and elevated (e[CO_2_]), error bars represent standard error. (**b**) Percent starch accumulation at e[CO_2_] by cultivar and FHB resistance score. Horizonal bars represent group mean starch accumulation, error bars represent standard error. Wheat cultivars were ordered by FHB resistance score at the top of the figure panel. (**c**) Percent starch accumulation by FHB group. Different letters or asterisks (*) denote statistically significant differences as determined by a Tukey adjusted generalized mixed model analysis of variance (*P* < 0.05); MR (*n* = 27), and susceptible (*n* = 18), for each CO_2_ growth condition.

While overall the percent change in protein content at e[CO_2_] was greater in the MR and there was no significant cultivar x [CO_2_] interaction, there were two notable exceptions in the trend, Rollag and Glenn. The percent change in protein content for Rollag and Glenn, which have a designated resistance score of 3, were on average comparable to the percent change shown for the susceptible cultivars with a score of 6 (Fig. 3b). Although Glenn, which had the greatest level of resilience in protein content, does not contain *Fhb1,* Bolles which similarly lacks *Fhb1* displayed above average change in protein content at e[CO_2_]. The exacerbated reduction in percent protein content of MR cultivars at e[CO_2_] was associated with greater starch accumulation (Figs. 3, 4). While there were differences in starch content among cultivars, the cultivar × [CO_2_] interaction was not significant. However, there was a significant interaction between the degree of FHB resistance × [CO_2_] (*P* = 0.014). Again, the susceptible cultivars had no significant change in starch content (3%) when grown at e[CO_2_], while the MR cultivars had significant accumulation (8%) of starch (*P* = 0.011; Fig. 4a). The percent change in grain starch content was correlated with increasing FHB resistance (*r* = 0.39; *P* = 0.0086; Fig. 4b,c). Furthermore, the overall percent change in starch accumulation at e[CO_2_] was greater in the MR compared with susceptible cultivars (*P* = 0.002; Fig. 3c). Changes in protein and starch at e[CO_2_] were both found to increase with FHB resistance score. Finally, starch and protein content were negatively correlated with one another at e[CO_2_] (*r* = − 0.59, *P* < 0.0001), suggesting that the loss of grain protein was due to dilution by the storage carbohydrate starch.

There were substantial differences in grain water soluble carbohydrate (WSC) content among wheat cultivars, but unlike starch, there was no significant effect of elevated CO_2_ on glucose, fructose, sucrose, or raffinose (supplemental Fig. 2). Only maltose was significantly higher (10%) at e[CO_2_] (*P* = 0.015), but there was no significant interaction between [CO_2_] and cultivar or FHB resistance. The total WSC content evaluated by HPAEC-PAD was not impacted by elevated CO_2._

**Figure 5:**
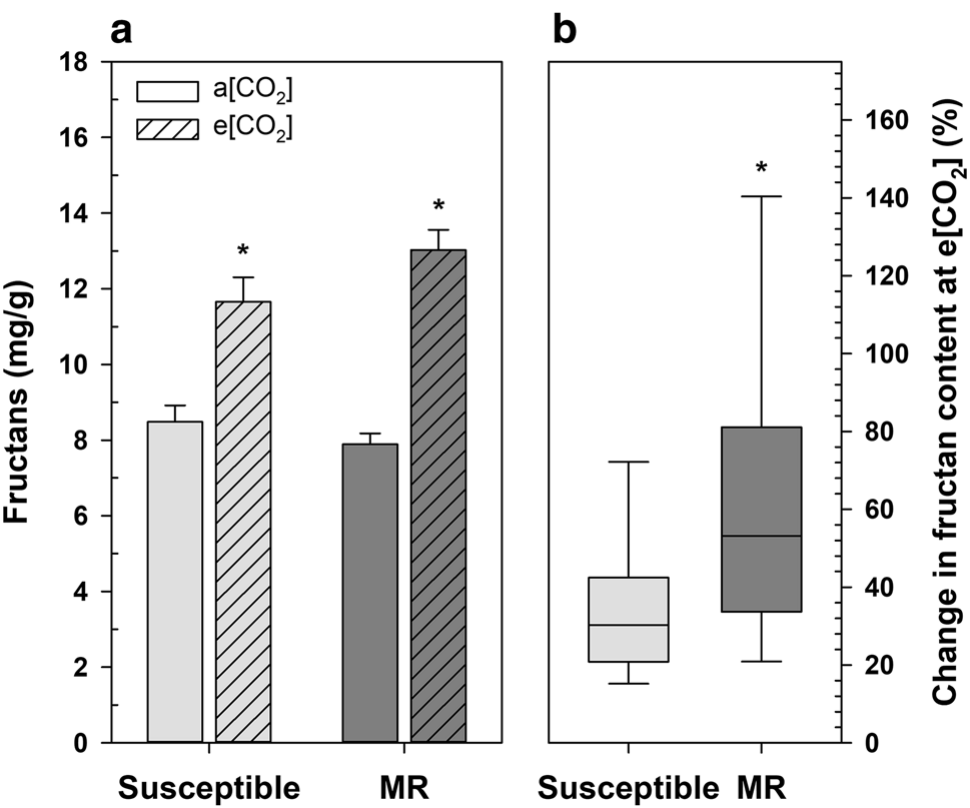
**(a**) Fructan concentration of susceptible and moderately resistant (MR) wheat grain grown at ambient CO_2_ (a[CO_2_]) and elevated CO_2_ (e[CO_2_]), error bars represent standard error. (**b**) Percent change in fructan content at e[CO_2_] by *Fusarium* head blight (FHB) resistance group. An asterisk (*) denotes a statistically significant effect of elevated CO_2_ on Fructan concentration as determined by Tukey adjusted generalized mixed model analysis of variance (*P* < 0.05); MR (*n* = 27), and susceptible (*n* = 18), for each CO_2_ growth condition.

Fructans are storage carbohydrates made up of polymeric fructose of varying chain length. Both MR and susceptible cultivars had higher fructan content at e[CO_2_] than at a[CO_2_] (*P* < 0.0001; Fig. 5a), but MR cultivars had a significantly greater percent change in fructan content (65%) compared with susceptible wheat (37%; Fig. 5b; *P* = 0.012).

Fatty acids are typically very minor components in wheat grain, but each fatty acid assayed substantially differed among cultivars. However, there was no significant impact of elevated CO_2_ on the ratios of fatty acids and there was no significant interaction between cultivar × [CO_2_] or FHB group (Supplemental Fig. 3). Palmitic acid was slightly higher in the susceptible cultivars at e[CO_2_] and lower in MR cultivars, but these differences were not statistically significant (*P* = 0.098).

**Figure 6:**
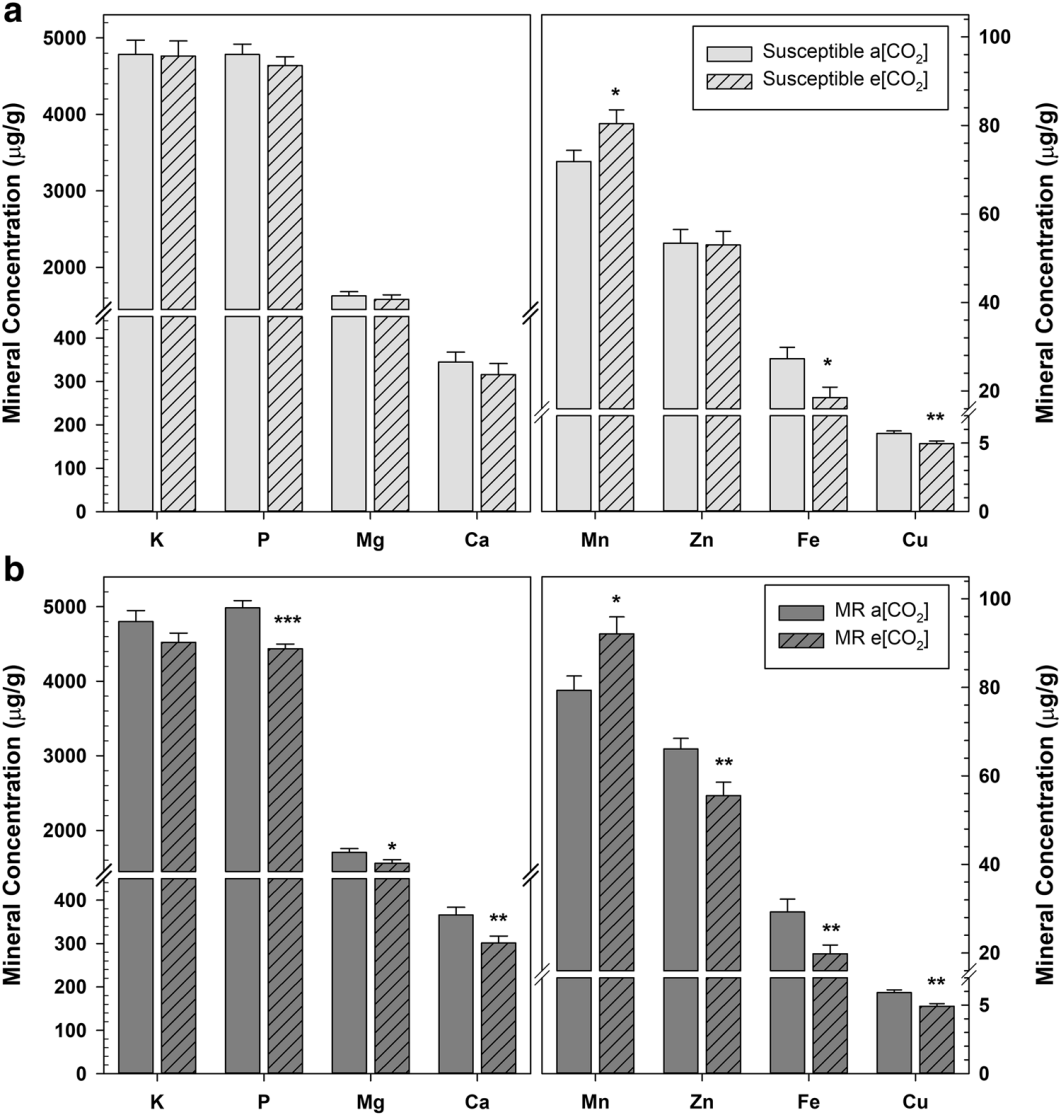
Mineral content of susceptible (**a**) and moderately resistant (MR) (**b**) wheat grain grown at ambient CO_2_ (a[CO_2_]) and elevated CO_2_ (e[CO_2_]). Error bars represent standard error and asterisks (*, **) denote statistically significant differences in the mineral content of wheat grown at a[CO_2_] and e[CO_2_] by *Fusarium* head blight (FHB) resistance group, as determined by a Tukey adjusted generalized mixed model analysis of variance (*P* < 0.05, *P* < 0.01, respectively); MR (*n* = 27), and susceptible (*n* = 18), for each CO_2_ growth condition.

Mineral nutrient content is essential for both plant growth and human nutrition; phosphorus, potassium, magnesium, calcium, manganese, zinc, iron, and copper are all defined as essential dietary nutrients^42^. Mineral content differed between cultivars and was significantly impacted by growth at e[CO_2_] (Fig. 6); however, there was no significant cultivar × [CO_2_] interaction. Growth at e[CO_2_] resulted in significantly less iron (32%) and copper (13%) in the susceptible cultivars (Fig. 6a; *P* < 0.01). The MR cultivars had less phosphorus (11%), magnesium (9%), calcium (18%), zinc (13%), iron (32%), and copper (17%) at e[CO_2_] (Fig. 6b; *P* < 0.01). There was a significant FHB resistance score × [CO_2_] interaction for phosphorus (*P* = 0.005) and magnesium content (*P* = 0.047); the greater a cultivar’s FHB resistance, the greater the difference in phosphorus (*r* = − 0.43; *P* = 0.0039) and magnesium (*r* = − 0.36; *P* = 0.0157) contents between ambient vs e[CO_2_] grown grain. Interestingly, there was more manganese content in both FHB susceptible (12%) and MR cultivars (16%) (Fig. 6; *P* = 0.0005) at e[CO_2_].

**Figure 7:**
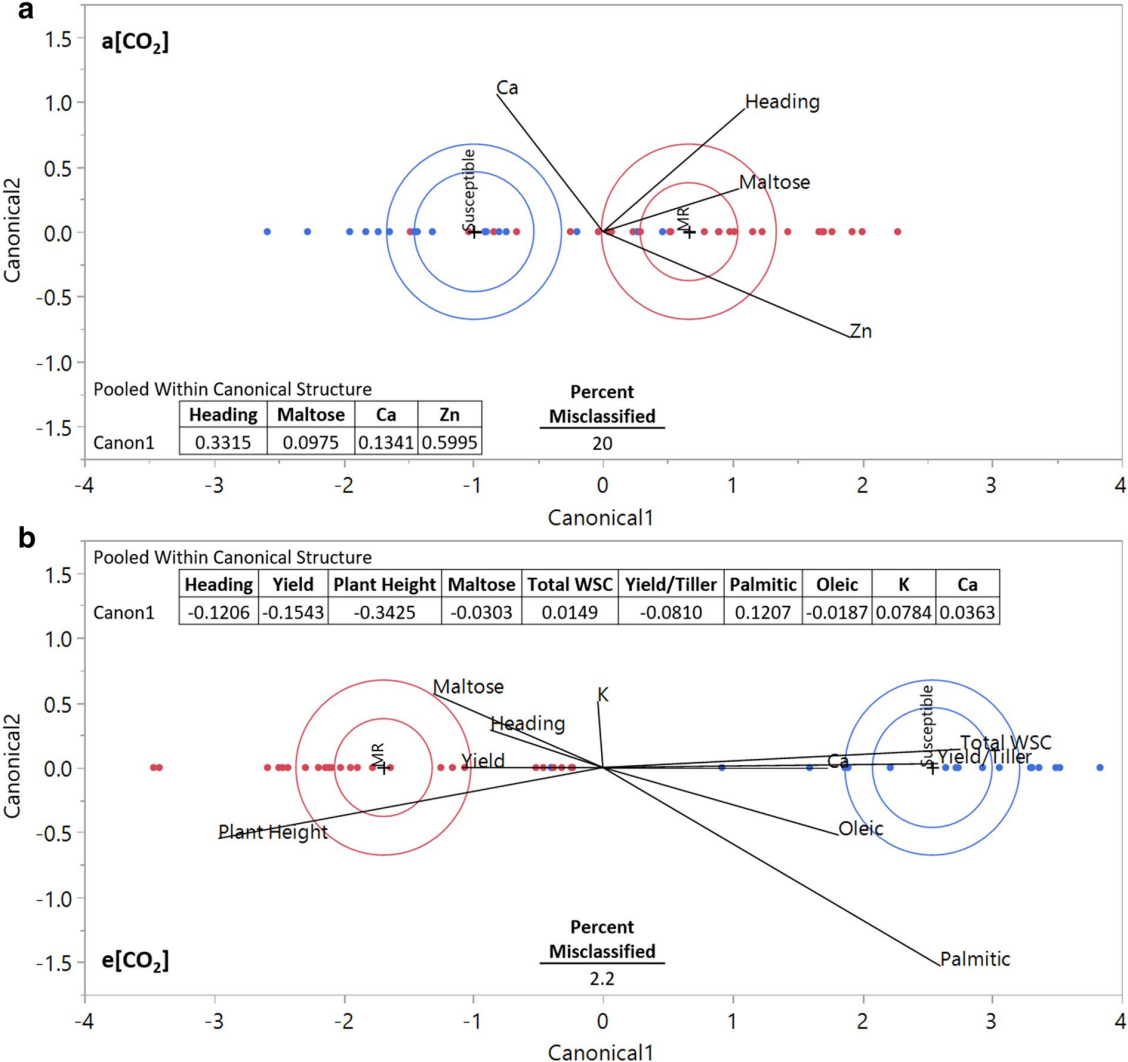
Discriminant analysis applied to the Fusarium head blight (FHB) susceptible and moderately resistant (MR) cultivars at (**a**) ambient CO_2_ (a[CO_2_]), and (**b**) elevated CO_2_ (e[CO_2_]). Groups are separated along Cannonical1 by the function coefficients of the independent variables presented in each table, found within the figure panel (**a,b)**. Blue objects represent susceptible cultivars, red MR. The inner circle represents the 95% confidence region of the group mean, and the outer circle represents the area containing 50% of the group population. Rays represent the independent variables relative association with the canonical variable along the Canonical1 axis.

After identifying numerous differences between FHB resistant groups at e[CO_2_] within the univariate generalized linear model approach, a multivariate discriminant analysis was performed to investigate separation of the variables as a collective entity. The analysis attempts to classify each cultivar to a given FHB group using the previously described results. The goal was to determine how well individual observations can be properly separated into groups and to identify the relative importance of key plant characteristics for FHB resistance level classification/identification. The function coefficients, presented in the Pooled Within Canonical Structure tables for each independent variable (Fig. 7), compare the relative association and influence of each independent variable in determining the discriminant score of the canonical variable (Canonical1).

At a[CO_2_], susceptible and MR groups were quite similar and difficult to differentiate, with a misclassification rate of 20% (Fig. 7a). The characteristics with the most discriminating power at a[CO_2_] were zinc, heading date, calcium, and maltose content, in order of relative importance. The eigenvalue at a[CO_2_], the amount of variance explained by the associated discriminant function, was only 0.7, and the entropy R^2^, the measure of fit, was 0.34; properly classifying cultivars into FHB resistance groups at current atmospheric conditions was prone to misclassification.

At e[CO_2_], susceptible and MR groups separated and were easily differentiated, with a misclassification rate of only 2% (Fig. 7b). The plant characteristics with the most discriminating power at e[CO_2_] were: plant height, yield per plant, heading date, palmitic acid, yield per tiller, potassium, maltose, calcium, oleic acid, and total WSC, in order of relative importance. The eigenvalue at e[CO_2_] was 4.5, with an entropy R^2^ of 0.87; a perfect fit has an entropy R^2^ of 1. The MR and susceptible cultivars were distinct from one another at e[CO_2_], and cultivars were accurately classified into FHB resistance groups. The high degree of group separation at e[CO_2_] (Fig. 7b) is consistent with the markedly different responses of MR and susceptible cultivars to growth at e[CO_2_]. The results of the discriminant analysis are consistent with the hypothesis of the study, showing that growth at e[CO_2_] more severely impacted FHB MR cultivars compared with susceptible.

## Discussion

In the present study, we demonstrated that the deleterious effects of elevated CO_2_ on wheat nutrition is more severe for FHB moderately resistant (MR) cultivars. To our knowledge, this is the first report of a correlation between the degree of plant pathogen resistance and grain nutritional content loss in response to elevated CO_2_. This is of significant importance because future wheat growers may be dissuaded from planting MR cultivars due to reduced end-use quality and marketability from growth at elevated CO_2_. Therefore, our results reveal the vital importance to identify wheat cultivars that can maintain nutritional integrity and FHB resistance in future climate conditions.

Currently, only a few QTLs have been validated to confer stable moderate FHB resistance, but no trait, or combination of traits, providing complete FHB resistance has been discovered^24,43^. The introgression of FHB resistance from Sumai 3 and other Chinese germplasm, the predominate sources of FHB resistance in North American wheat, may have resulted in the unintended reduction of nutritional content, which is further exacerbated at e[CO_2_].

Reduced grain protein has also been associated with the introgression of the FHB resistance QTLs *Fhb1* and *Fhb5,* as well as the stacking of other *Fhb* QTLs; these decreases in protein content were found to be dependent on the donor and recipient genetic background^25,26^. However, sources of *Fhb1* from various Chinese donor cultivars were found to have no negative impact on agronomic traits in winter wheat, but the degree of FHB resistance in the recipient cultivar varied significantly, likely due to additional minor QTLs introgressed from the donor^44^. Improved FHB resistance is also associated with a number of minor loci from resistant donor lines, mostly genes expressing a variety of plant defense related kinases, nucleotide-binding and leucine rich repeats^45,46^. The introgression of these disease resistant traits from non-adapted donors can cause significant linkage drag, negatively affecting agronomic traits^46,47^. Nevertheless, it will be essential for breeders to know if *Fhb1* is associated with the more severe loss in grain protein content at elevated CO_2_. The limited number of MR cultivars without *Fhb1* used in this study does not allow for accurate correlations between the *Fhb1* QTL and the quality penalty under elevated CO_2_. In future studies we are addressing this critical question utilizing near isogenic lines with and without the *Fhb1* QTL. It may be necessary to use FHB resistance genes not derived from the Sumai 3 cultivar, such as *Fhb7*, which encodes a glutathione-S-transferase to detoxify trichothecene mycotoxins^43^. This recently identified source of resistance may provide plant breeders an alternative method of protecting wheat from FHB while improving nutritional climate resilience.

Disease resistance traits often have trade-offs, negative pleiotropic effects on important agronomic characteristics such as yield, nutritional quality, and resistance to other diseases or pests^48^. The allocation of plant resources for self-protection necessarily reduces available resources for growth or reproduction, and even the presence of resistance genes generally results in a loss of fitness^49^. The decline of grain nutritional content in the MR cultivars at elevated CO_2_ may be due to this defense trade-off paradigm, particularly as FHB resistance is often tightly associated with traits which reduce crop performance and grain quality, and the introgression of these traits may enhance disease resistance at the cost of reduced yield and less grain protein^26,50^.

However, breeding efforts currently manage to maintain acceptable gain quality standards in MR cultivars. Rising CO_2_ may exacerbate the negative effects on agronomic traits, making cultivar selection to maintain grain quality and FHB resistance more difficult in the future. The significant decrease in grain protein content at e[CO_2_] in the MR cultivars (Fig. 3) is highly concerning, as FHB resistance traits may become a nutritional liability in future climate conditions. Farmer cultivar selection is typically focused on yield, grain quality, loss avoidance, and marketability. If MR cultivars have declining grain quality and marketability with rising CO_2_, farmers may abandon disease resistant lines for more susceptible cultivars, significantly increasing the risk of FHB outbreaks.

Furthermore, elevated atmospheric CO_2_ may increase MR wheat susceptibility to FHB and mycotoxin contamination due to the substantial changes in nutrient composition. Starch is a known inducer of trichothecene mycotoxins by Fusarium fungal pathogens, and changes in grain nutrient composition at elevated CO_2_ have been observed to increase pathogen trichothecene mycotoxin production^10,51^. Furthermore, virulence-associated genes are often linked to major nitrogen regulatory transcription factors and toxin production is considerably influenced by the available nitrogen of the host^52,53^. However, FHB resistance is also significantly correlated with increased plant height^54–56^, thus further research is needed to empirically determine the full effects of these physiological changes on host susceptibility to FHB.

Previous reports have shown that wheat grown at elevated CO_2_ has higher grain carbohydrate content and lower relative amounts of proteins, minerals, and lipids^13,14^. The decline of grain protein in the MR cultivars (Fig. 3) was directly correlated with the accumulation of storage carbohydrates, starch, and fructans (Figs. 4, 5), which is consistent with the dilution effect of excess carbohydrates. Increased nitrogen application may reduce these dilution effects. However, complex biological partitioning mechanisms are also likely involved^6,13,15,16^. The uniform decrease in iron concentrations across all FHB groups grown at e[CO_2_], regardless of starch accumulation, suggests a more complex metabolic response.

The observed increased grain yield per plant at elevated CO_2_ (Fig. 2) was consistent with previous reports^57,58^. Additionally, global wheat yields are predicted to significantly increase with rising CO_2_, though these gains are highly dependent on growing temperatures, water availability, and nitrogen fertilization^5,12,59^. Furthermore, typical high planting densities in wheat may increase plant height and lodging risk due to increased intra and inter-plant competition^60^. Increased nitrogen fertilization to maintain grain protein and improve yield at elevated CO_2_ was found to be only somewhat effective; however, increased nitrogen fertilizer rates did increase internode length, plant height, and ultimately lodging risk^61,62^. Attempts to ameliorate grain nutritional losses with increased fertilization may simply lead to more lodging, thus a better focus may be identifying climate resilient wheat cultivars.

Increased wheat plant height can lead to a greater risk of crop lodging, stem bending and crop collapse, which can substantially reduce yields and lead to fungal contamination, but is highly dependent on cultivar and planting density^63^. Plant height dramatically increased in the MR cultivars (Fig. 1) and was the most discriminating characteristic for defining FHB groups at e[CO_2_] (Fig. 7). Furthermore, wheat stem strength was lower in MR cultivars RB07 and Bolles at e[CO_2_], potentially exacerbating the risk of crop lodging. The current study is inherently limited in fully characterizing the impact of elevated CO_2_ on wheat in future field conditions. Growth in enclosed chambers can alter and downregulate photosynthesis, stifle root structure and volume, and the lack of wind induced mechanical stress may change the rate of lignin deposition^64,65^. Further research at a free air carbon enrichment (FACE) facility is needed to elucidate cultivar specific lodging risk at elevated CO_2_.

The loss of grain protein at elevated CO_2_ is not simply an issue of human nutrition. The end use suitability of wheat flour is intractably linked to grain protein content. Low protein flour (8–11%) is generally used for cakes and pastries while high protein flours (> 14%) are used for breads and pastas^5,66^. Wheat grown at elevated CO_2_ often has significantly less grain protein, which reduces the baking quality, compromising the final food product^7,17,18^. Increased starch content at elevated CO_2_ has also been associated with reduced baking quality as well as starch damage during the milling process^7^. Future farmers and food processors can anticipate substantially increased wheat yields of lower quality grain with poorer dough rheology, reduced loaf volume and baking quality^19^. The identification of wheat cultivars that can maintain nutritional integrity and FHB resistance in future climate conditions are critically needed to ensure the safety and security of our food supply.

## Supplementary Information

Supplementary Information.

## Data Availability

The datasets generated during and/or analyzed during the current study are available from the corresponding author on reasonable request.
