# Supplementary material for: Fusarium head blight resistance exacerbates nutritional loss of wheat grain at elevated CO2
Source: Sci Rep. 2022 Jan 7;12:15. doi: 10.1038/s41598-021-03890-9 (PMC8741757; doi:10.1038/s41598-021-03890-9)
Supplement: Supplementary file 1 — Supplementary Information. [file 41598_2021_3890_MOESM1_ESM.pdf]

TITLE: Fusarium head blight resistance exacerbates nutritional loss of wheat grain at elevated CO_2

Supplemental:

Cultivar						Heading (days)
MN08173-3-10	A					47.8
MN11394-6	A	B				47.2
Bolles	A	B	C			46.2
MN11492-6	A	B	C			45.5
Shelly	A	B	C			45.0
Lang-MN	A	B	C			44.8
Wheaton	A	B	C			44.8
MN00269	A	B	C			44.3
RB07		B	C	D		44.2
Linkert		B	C	D		43.7
Rollag			C	D	E	43.5
Sabin			C	D	E	43.5
MN10281-1-98				D	E	40.7
Glenn					E	40.0
Ulen					E	40.0

Cultivar							Flowering (days)
MN08173-3-10	A						53.0
Wheaton	A	B					51.5
MN11394-6	A	B					51.3
Bolles	A	B	C				50.5
MN00269		B	C	D			49.7
MN11492-6		B	C	D			49.7
Shelly		B	C	D			49.7
Lang-MN		B	C	D			48.8
RB07		B	C	D			48.8
Rollag		B	C	D			48.8
Linkert			C	D	E		47.8
Sabin				D	E	F	46.7
Glenn					E	F	45.0
Ulen						F	44.3
MN10281-1-98						F	43.7

Supplemental Table 1. Wheat developmental timings, days after planting, for heading and flowering by cultivar. Different letters denote significant differences as determined by a Tukey adjusted generalized mixed model ANOVA ($\alpha = 0.05$; JMP V15.0).

Cultivar	CO2		Mean Height (cm)
MN08173-3-10	e[CO2]	A	95.18
Glenn	e[CO2]	A B	88.35
Sabin	e[CO2]	B C	83.44
Lang-MN	e[CO2]	B C D	82.40
Ulen	e[CO2]	B C D E	80.98
MN11394-6	e[CO2]	B C D E F	80.44
MN08173-3-10	a[CO2]	B C D E F G	79.97
Bolles	e[CO2]	B C D E F G H	78.59
RB07	e[CO2]	C D E F G H I	74.65
Glenn	a[CO2]	C D E F G H I	74.46
Ulen	a[CO2]	D E F G H I J	72.80
Lang-MN	a[CO2]	E F G H I J	71.27
Shelly	e[CO2]	E F G H I J	71.23
Wheaton	e[CO2]	E F G H I J K	70.46
Rollag	e[CO2]	F G H I J K	70.40
MN11394-6	a[CO2]	G H I J K	69.78
Sabin	a[CO2]	G H I J K	69.53
Linkert	e[CO2]	H I J K L	68.47
MN10281-1-98	e[CO2]	H I J K L	68.41
Bolles	a[CO2]	I J K L	67.79
MN00269	e[CO2]	I J K L	66.92
RB07	a[CO2]	I J K L	65.93
Rollag	a[CO2]	I J K L	65.85
Wheaton	a[CO2]	I J K L	65.82
MN11492-6	e[CO2]	I J K L	65.49
MN10281-1-98	a[CO2]	J K L	63.45
Linkert	a[CO2]	J K L	63.40
MN11492-6	a[CO2]	J K L	62.63
MN00269	a[CO2]	K L	60.56
Shelly	a[CO2]	L	58.86

Supplemental Table 2. Significant differences in plant height as determined by wheat spike height (cm) at ambient (a[CO_2]) and elevated (e[CO_2]) carbon dioxide concentration. Different letters indicate significant differences as determined by a Tukey HSD ($\alpha = 0.05$) JMP 15.0.

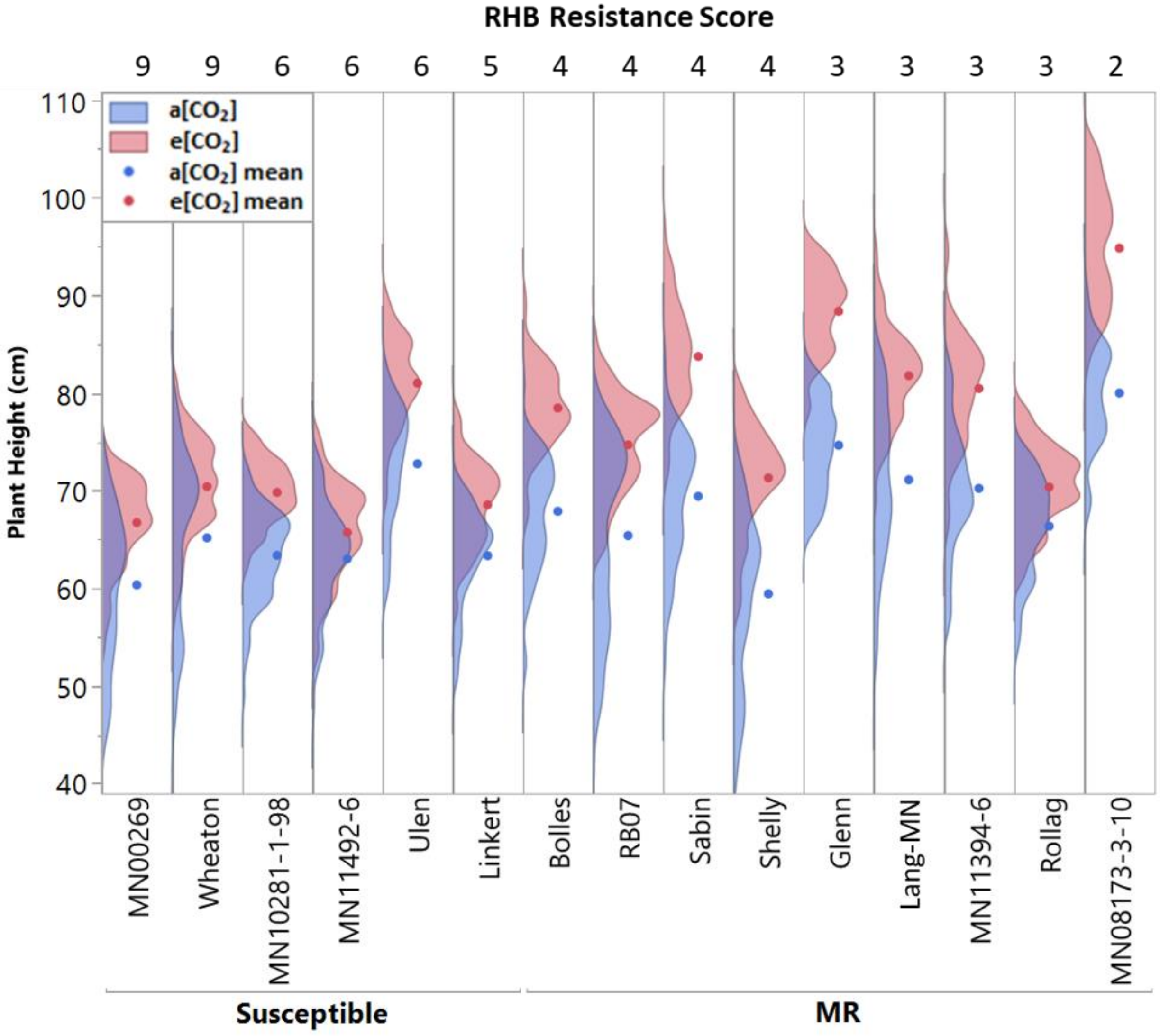

Supplemental Figure 1. Histogram of plant height of various wheat cultivars at Feekes 11.3. Blue and red areas represent the percent distribution of tiller heights at ambient CO_2 (a[CO_2]) and elevated CO_2 (e[CO_2]), respectively. Wheat cultivars were ordered by FHB resistance score at the top of the figure panel. Blue and red points represent mean plant height (cm) for cultivars at ambient CO_2 or elevated CO_2, respectively.

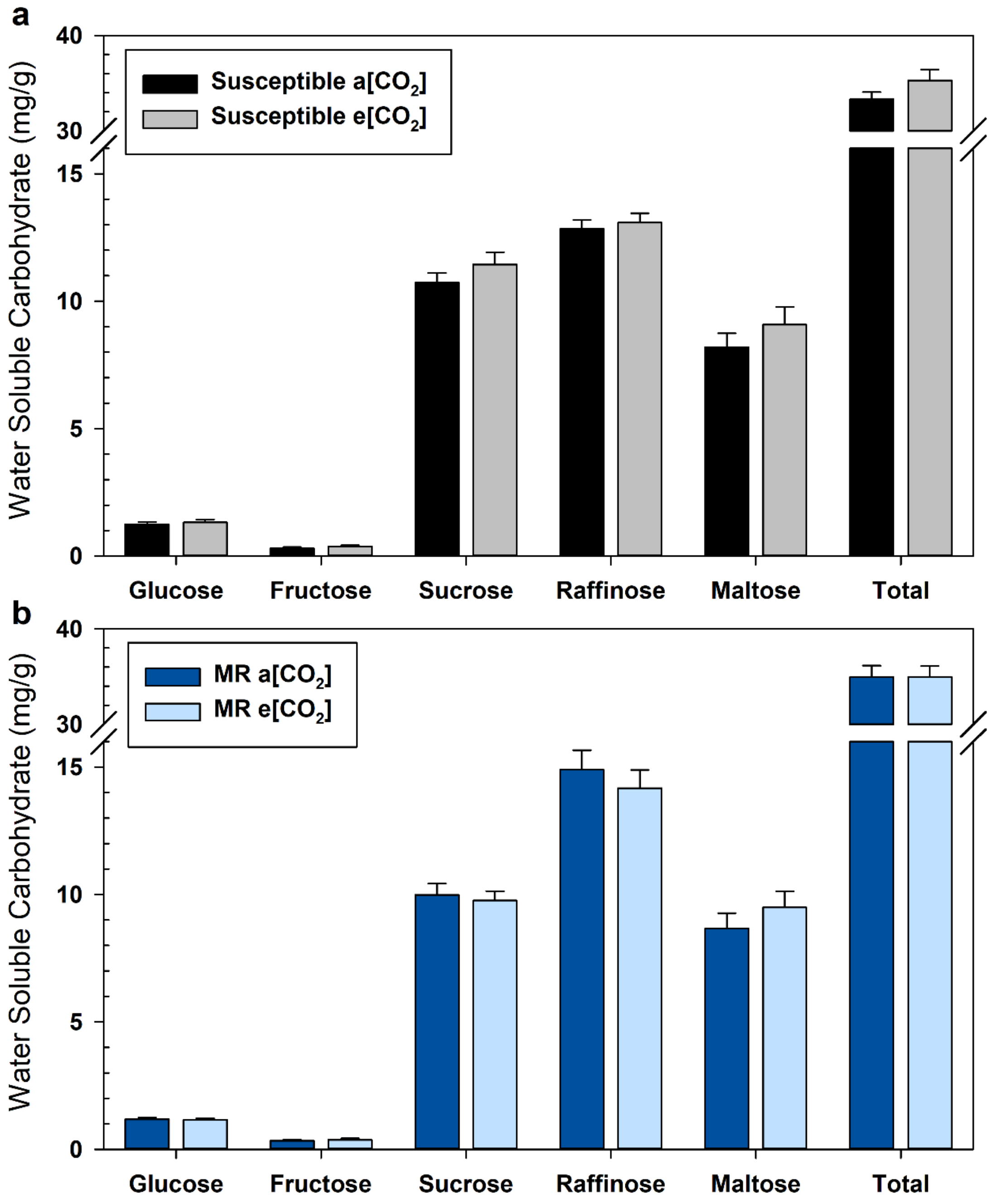

Supplemental Figure 2. Grain water soluble carbohydrates extracted from susceptible (**a**) and MR (**b**) cultivars grown at either ambient (a[CO_2]) or elevated CO_2 (e[CO_2]). Error bars represent the standard error of the mean.

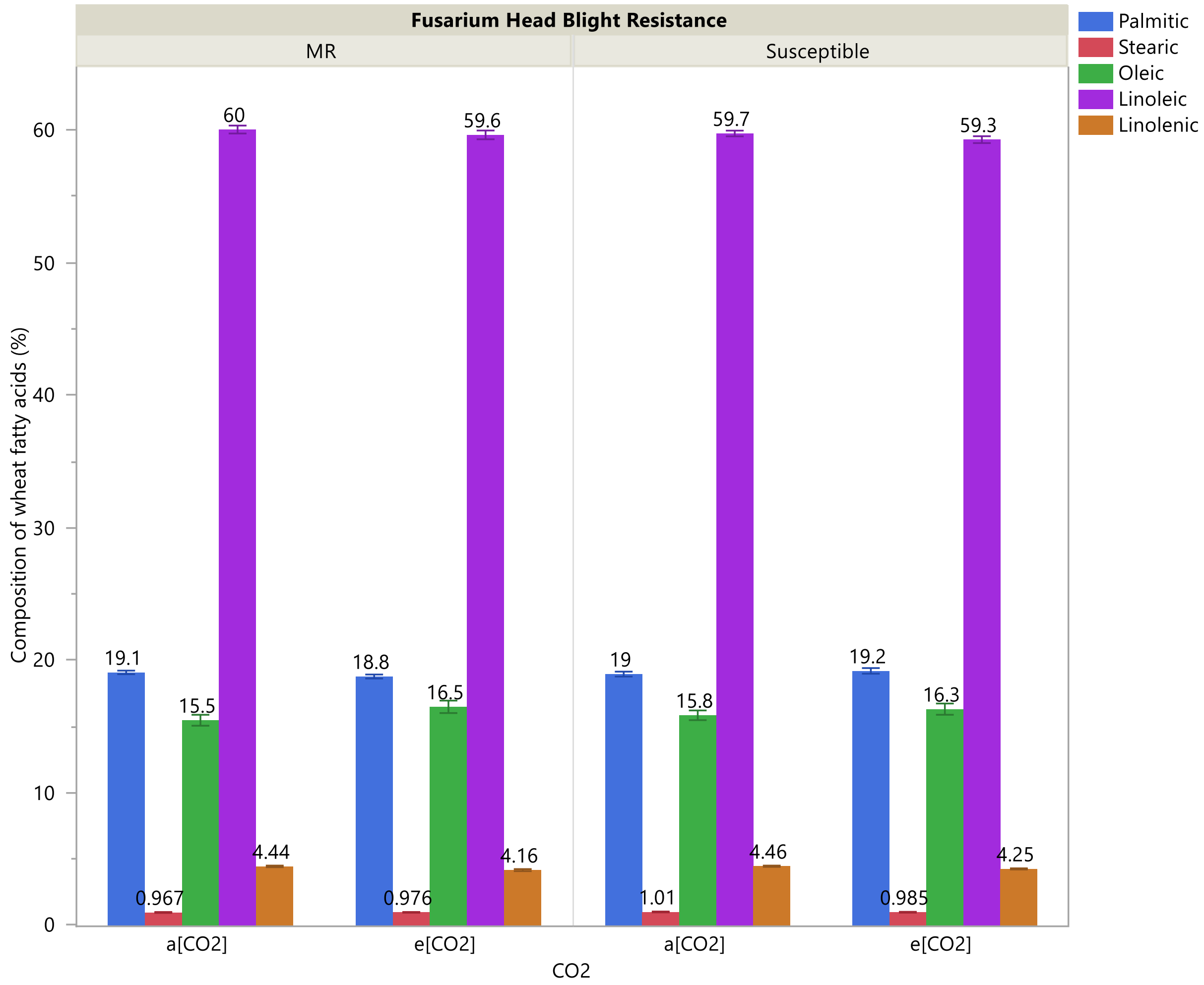

Supplemental Figure 3. Composition of total fatty acids extracted from MR and susceptible wheat grain grown at ambient (a[CO_2]) and elevated CO_2 (e[CO_2]). Error bars represent the standard error of the mean.
